# Influence of Consanguinity on Non-communicable Diseases in Settat, Morocco: Exploring Susceptibility to Chronic and Complex Health Conditions

**DOI:** 10.34172/jrhs.2025.181

**Published:** 2025-04-01

**Authors:** Khaddouj El Goundali, Milouda Chebabe, Noureddine Elkhoudri, Abderraouf Hilali

**Affiliations:** ^1^Laboratory of Health Sciences and Technologies, Settat, Morocco; ^2^Higher Institute of Health Sciences, Hassan First University of Settat, Settat, Morocco

**Keywords:** Non-communicable diseases, Consanguinity, Cancer, Diabetes, Asthma, Cardiovascular diseases

## Abstract

**Background:** The prevalence of non-communicable diseases (NCDs) represents a significant global health challenge, accounting for 71% of global deaths. This concern is also widespread in Morocco. Consanguinity, common in Arab and Muslim regions, could influence the genetic predisposition to these diseases. This study aimed to explore the impact of consanguineous marriage (CM) on predisposition to these chronic illnesses among the populace in Settat province, Morocco, concerning prevalent NCDs.

**Study Design:** A cross-sectional study.

**Methods:** This study was conducted in Settat, Morocco, from April to October 2021 and included 453 married women aged 18 and above. Participants were selected from rural and urban health centers using a two-stage sampling method. The data were collected through structured interviews using a validated questionnaire. Statistical analyses with SPSS 26 assessed associations between consanguinity and NCDs in respondents and their descendants using odds ratios (ORs) and 95% confidence intervals.

**Results:** The participants had a mean age of 38.04 years, with 35% residing in rural areas and 26.7% having a CM. The results indicated that individuals with consanguineous ancestors have a greater risk of contracting NCDs, such as cardiovascular diseases (CVDs) (OR=2.047, *P*=0.005), diabetes (OR=1.988, *P*=0.009), asthma (OR=2.069, *P*=0.036), chronic kidney disease (CKD) (OR=1.732, *P*=0.045), and cancer (OR=1.646, *P*=0.1), compared to those with non-consanguineous parents.

**Conclusion:** It is essential to integrate the harmful effects of consanguinity on future generations’ health into public health policy through genetic counseling, testing, screening, and awareness programs.

## Background

 Non-communicable diseases (NCDs) are considered the most pressing public health issue, representing the 21st century’s affliction and a challenge for all countries. Among a total of 57 million global deaths, 71% are attributed to NCDs, with 15 million premature deaths occurring between the ages of 30 and 70.^1^ They are increasing exponentially and pose a threat to global economic growth and development. Studies recently published by the World Economic Forum and Harvard School of Public Health have projected that within the coming fifteen years, NCDs will incur a financial toll of over $7 trillion in national income losses for low- and middle-income countries. This observation urges countries to urgently implement measures to improve population health and prevent NCDs.^2^ In Morocco, the situation is as concerning as it is globally. The shift in epidemiological and demographic patterns results in a heightened burden of NCDs in terms of both illness rates and mortality, particularly impacting conditions such as cardiovascular diseases (CVDs), chronic respiratory diseases, diabetes, cancer, and chronic kidney disease (CKD).^2^ These illnesses stand as the primary cause of death, representing 80% of all fatalities, positioning Morocco among the nations in the Eastern Mediterranean region with high mortality rates attributed to NCDs.^1^

 These complex diseases display substantial diversity in their origins. They entail a hereditary component in most familial instances and individual cases.^3^ Genes that demonstrate heightened susceptibility might substantially contribute to the onset of multifactorial diseases. In cases where these genes are rare and inherited through an autosomal recessive inheritance pattern, consanguineous marriages (CMs) might become a contributing factor.^4^ CMs are widespread in various regions, particularly among Muslim populations in North Africa, the Middle East, and South Asia. These marriages constitute a significant portion of unions, affecting over a billion people worldwide, with rates ranging from 20% to 70% depending on the regions.^4-8^ The highest rates are found in the Arab world, notably in Saudi Arabia (50.5%)^9^, Qatar (54%),^6^ Oman (1.6%),^10^ and Kuwait (37.7-56.3%),^11^ sometimes reaching 86.6%.^12^ Several socio-economic, sociocultural, religious, geographical, and demographic factors have been identified as influencing these rates, but these determinants vary across countries and regions.^13-15^

 Most research has concentrated on investigating the influence of CM on the health and survival of infants. Nevertheless, there is insufficient information on the possible involvement of consanguinity and recessive genes in prevalent NCDs,^4^ which in turn may indirectly contribute to adverse reproductive health outcomes.^14^

 Despite its negative health implications, CM remains prevalent in Morocco, as in many other Arab and Muslim nations, with a prevalence rate of 23.4%.^16^ Simultaneously, public health concerns about genetic diseases as significant contributors to morbidity and mortality are becoming more prominent, especially with the decline in infectious diseases.^4^ It is against this global backdrop that Morocco’s national multisectoral strategy for the prevention and control of NCDs (2019-2029) is positioned. This strategy emanates from the commitment of United Nations member states to prioritize the prevention and management of NCDs within the framework of sustainable development.^1^ From this standpoint, the present study investigates the influence of CM among the populace in Settat province, Morocco, concerning prevalent NCDs. It specifically targets vulnerability to various chronic and intricate conditions such as diabetes, CVDs, cancer, asthma, and CKD.

## Methods

###  Study setting

 This is a cross-sectional analytical study based on a survey conducted in the Settat province (Morocco). The study spanned seven months, conducted from April to October 2021. Settat province, situated at the heart of the Kingdom, is within the Casablanca-Settat mega-region, known as the nation’s primary multi-sector economic center. Covering around 7220 km^2^, it accounts for 35% of the regional area.^17^

###  Population and sampling plan

 Participants were eligible to participate in the study if they were married women aged 18 years and older, agreed to take part in the study, and were of Moroccan origin. The study sample was determined using a two-step sampling approach. The first step involved randomly selecting two primary healthcare services from the Settat region, which covered both urban and rural areas. A convenience sampling method was used in the second step. All women who met the inclusion criteria and attended the study sites during the data collection period were included in the sample. This approach was chosen for its effectiveness in achieving the required number of participants within the available time frame.

###  Sample size 

 The sample size was determined based on a confidence level of 95%, an estimated prevalence of consanguinity among married women of 23.4% (*P* = 0.234), and a margin of error of 5%. To account for potential non-responses, a 20% non-response rate was incorporated, resulting in a calculated sample size of 272 participants. In the field, 548 women were approached, of whom 453 agreed to participate, yielding a response rate of 82.66%, which exceeded the required sample size.

###  Data collection

 The questionnaire used in this study was designed by the authors based on a relevant literature review. It was then validated by laboratory experts and members of the ethics committee, who assessed its content validity (content validity ratio) and cultural relevance. Face validity was tested on 30 women speaking the local language, which led to revisions of ambiguous questions for clarity. Reliability was confirmed with a Cronbach’s alpha of 0.724, indicating an acceptable internal consistency of the questionnaire.

###  Questionnaire structure

1. *Socio-demographic variables*: Age, marital status, place of residence, and level of education
2. *Socio-economic variables*: Profession (respondent and spouse) and household income
3. *Anthropological variables*: Type of marital alliance (consanguineous or non-consanguineous) and degree of relationship (first cousins or distant cousins)
4. *Health-related variables*: Presence of NCDs (diabetes, CVDs, asthma, CKD, and cancer)

###  Implementation and bias control

 The required data were collected through structured, face-to-face interviews conducted by trained healthcare professionals fluent in the local language to ensure accurate and reliable responses. To minimize biases, multiple sources of information (e.g., prescriptions and health records) were utilized to reduce recall bias. Incomplete questionnaires were excluded from the investigation. The questionnaire used for the survey was designed and validated by laboratory experts, ensuring both content and apparent validity.

###  Statistical analysis

 The data were collected, coded, and tabulated using SPSS 26. The analysis involved univariate and bivariate techniques. Odds ratios (ORs) and 95% confidence intervals (CIs) were calculated to assess the susceptibility to NCDs based on inbreeding status, both in the current generation and among the respondents’ descendants. In this analysis, individuals from CM were considered cases, while individuals from non-CM were regarded as controls. The same categorization was applied when assessing the respondents’ descendants. To examine relationships between categorical variables, the Chi-square test was used, with statistical significance set at a *P* value of < 0.05.

###  Marriage categorization and consanguinity coefficient

 Marriages were categorized based on the degree of biological relationship between spouses, with the following classifications:

- Double first cousins (F = 1.8): Sharing both sets of grandparents
- First cousins (F = 1.16): Sharing one set of grandparents
- First cousins once removed (F = 1.32): One cousin having children with a cousin from another generation
- Second cousins (F = 1.64): Sharing great-grandparents
- Unrelated marriages (F = 0): Having no shared genetic material

The mean consanguinity coefficient was calculated using the following formula:

 α = ΣPiFi\alpha = \Sigma Pi Fiα = ΣPiFi

 where Fi represents the consanguinity coefficient for each category of CM, and Pi denotes the proportion of individuals in each category within the population. This formula helps calculate the average level of genetic relatedness within the study population based on the different degrees of consanguinity.^4,18^

## Results

###  Characteristics of the studied population

 The study included 453 women, most of whom were married (97.35%). The majority of them were aged between 40-49 (31.57%) and 20-39 (49.01%) years. In terms of education, 30.24% had no formal education, while 12.59% attained university-level qualifications. Most participants resided in urban areas (65.12%) and were not engaged in formal employment (87.41%). Household income levels varied, with 39.96%, 42.60%, and 17.44% classified in the low (earning less than 2800 MAD), middle (2800-6763 MAD), and high (exceeding 6763 MAD) economic levels, respectively (Table 1).

**Table 1:** Characteristics of participants

**Characteristics**	**Frequency**	**Percent**
Marital status		
Married	441	97.35
Divorced	7	1.55
Widowed	5	1.10
Age (year)		
< 20	9	1.99
20-29	115	25.38
30-39	107	23.63
40-49	143	31.57
50-59	67	14.79
≥ 60	12	2.65
Educational level		
No education	137	30.24
Primary	130	28.69
Preparatory	50	11.03
Secondary	79	17.44
University	57	12.59
Place of residence		
Urban	295	65.12
Rural	158	34.88
Occupation		
Employee	16	3.53
Public servant	31	6.84
Liberal profession	14	3.09
Day laborer	9	1.99
Other	2	0.44
No activity	396	87.41
Household income (MAD)		
< 2800	181	39.96
2800-6763	193	42.60
> 6763	79	17.44

*Note*. MAD: Moroccan dirham.

###  Consanguineous marriage in the province of settat

 In the studied population, the observed consanguinity rate was 26.7%, with an average coefficient of consanguinity of 0.0145871. Among the various forms of CM, cousin unions prevailed as the most common, representing 18.5% of all marriages and constituting 69.4% of all CMs in the Settat province. Then, first-degree distant cousins accounted for 2.6%, succeeded by second-degree distant cousins (2.2%), first-degree half cousins (1.8%), and the least prevalent type being double first-degree cousins (1.5%), the details of which are provided in Table 2.

**Table 2:** Categorization of CM in the settat province based on the level of consanguinity

**Degree of consanguinity**	**Frequency**	**Percent**	**Consanguinity coefficient**
Double first cousin	7	1.5	0.0019
First cousin (including all types):	84	18.5	0.0115
Patrilateral parallel cousins	30	6.6	
Matrilateral parallel cousins	32	7.1	
Cross-cousins: father’s sister’s son	12	2.6	
Cross-cousins: mother’s brother’s son	10	2.2	
First cousin once removed	12	2.6	0.0008
Second cousin	8	1.8	0.0002
Less than second cousin	10	2.2	
No relation	332	73.3	
Total	453	100	

 The comparison of CM rates between the current generation and the parents’ generation revealed that consanguinity was significantly more prevalent in the current generation for women (26.7% versus 18.5%, *P*= 0.019). It was also higher than that observed in the parents of the husbands (26.7% versus 14.8%, *P*= 0.000). Moreover, consanguinity coefficients were higher in the studied couples’ generation compared to their parents’ generations, with consanguinity coefficients of 0.0145871, 0.008392, and 0.0073601 in the studied couples’ generation, the women’s parents’ generation, and the husbands’ parents’ generation, respectively (Table 3).

**Table 3:** Prevalence of consanguinity in the present generation in contrast to the previous parental generation

**Degree of consanguinity**	**Current generation**	**Parents of wife**	**Parents of husband**
Double first cousin	7	4	2
First cousin	84.5	43	44
First cousin once removed	12.6	16	9
Second cousin	8	7	5
Lessthan second cousin	10	14	7
Coefficient of consanguinity	0.014	0.008	0.007

###  Non-communicable diseases and consanguinity

 In the context of this study, out of a sample of 453 women, 84 came from CM, representing 18.5% of the sample. The data revealed a higher prevalence of NCDs among participants from consanguineous families. The rate of diabetes was 32.1% among individuals from these marriages compared to 19.2% among those from non-CM. As regards CVDs, the corresponding percentage was 36.9% among individuals from consanguineous families, as opposed to 22.2% among those from non-consanguineous families. All other reported health conditions were more frequent among descendants of CM, including asthma (15.5% versus 8.1%), chronic renal failure (8.3% versus 2.4%), and cancer (11.9% versus 7.6%).

###  Consanguinity and predisposition to chronic diseases

 Based on the results, respondents from consanguineous parents showed a significantly higher risk compared to those from non-consanguineous parents (Table 3). They were twice as likely to have a communicable disease such as CVDs (OR = 2.047, 95% CI: 1.234–3.398, *P* = 0.005), diabetes (OR = 1.988, 95% CI: 1.175–3.364, *P* = 0.009), and asthma (OR = 2.069, 95% CI: 1.028–4.163, *P*= 0.036). They were almost four times more likely to have CKD (OR = 3.636, 95% CI: 1.314–10.063, *P* = 0.016). Regarding cancer, consanguineous individuals had a 1.6 times higher risk of developing this disease compared to their non-consanguineous counterparts (OR = 1.646, 95% CI: 0.766–3.535, *P*= 0.1). The related data are summarized in Table 4.

**Table 4:** Possible impact of consanguinity on susceptibility to NCDs in Settat province

**Variables**	**Consanguineous** **marriages (n=84)**		**Non-consanguineous** **marriages (n=369)**		**OR (95% CI)**	***P*** ** value**
	**Number**	**Percent**	**Number**	**Percent**		
Diabetes						
No	57	67.9	298	80.1	1.000	
Yes	27	32.1	71	19.2	1.988 (1.175–3.364)	0.009
CVD						
No	53	63.1	287	77.8	1.000	
Yes	31	36.9	82	22.2	2.047 (1.234–3.398)	0.005
Asthma						
No	71	84.5	339	91.9	1.000	
Yes	13	15.5	30	8.1	2.069 (1.028–4.163)	0.036
CKD						
No	77	91.7	360	97.6	1.000	
Yes	7	8.3	9	2.4	3.636 (1.314–10.063)	0.016
Cancer						
No	74	88.1	341	92.4	1.000	
Yes	10	11.9	28	7.6	1.646 (0.766–3.535)	0.100

*Note*.NCD: Non-communicable disease; CI: Confidence interval; OR: Odds ratio; CKD: Chronic kidney disease; CVD: Cardiovascular diseases.

###  Chronic diseases in descendants of studied couples

 Our findings demonstrated a notable link between consanguinity and NCDs among the offspring of the examined couples, specifically in relation to diabetes (*P* = 0.001), cancer (*P* = 0.013), and asthma (*P* = 0.013). Non-consanguineous individuals exhibited an 11% lower risk of diabetes, a 30% decreased risk of asthma, and a 17% reduced risk of cancer (Table 5).

**Table 5:** Non-Communicable Diseases Among the Offspring of the Studied Couples

**Variables**	**Consanguineous** **marriages (n=121)**		**Non-consanguineous** **marriages (n=332)**			
	**Number**	**Percent**	**Number**	**Percent**	**OR (95% CI)**	***P*** **-value**
Diabetes						
No	112	92.6	329	99.1	1.000	
Yes	9	7,4	3	0.9	0.113 (0.030–0.427)	0.001
CVD						
No	116	95.9	325	97.9	1.000	
Yes	5	4.1	7	2.1	0.500 (0.156–1.605)	0.192
Asthma						
No	111	91.7	323	97.3		
Yes	10	8.3	9	2.7	0.309 (0.123–0.781)	0.013
CKD						
No	120	99.2	329	99.1	1.000	
Yes	1	0.8	3	0.9	0.094 (0.113–10.621)	0.709
Cancer						
No	115	95	329	99.1	1.000	
Yes	6	5	3	0.9	0.175 (0.043–0.710)	0.013

*Note*. CI: Confidence interval; OR: Odds ratio; CKD: Chronic kidney disease; CVD: Cardiovascular diseases.

## Discussion

 The results of this study confirmed a high prevalence of CM in the Settat province (26.7%), which exceeds the national average of 23.4%^16^. The prevalence of CM in the current generation was significantly higher than that observed in the participants’ parents, highlighting that this practice remains deeply rooted in cultural and social traditions and could have significant implications for public health and preventive policies.

 The findings of this study identified a significant association between CM and NCDs. Individuals from CM showed an increased risk of CVDs, diabetes, asthma, CKD, and cancer. These findings are consistent with those of several international studies. Overall, most research supports an association between consanguinity and an elevated risk of NCDs, although variations exist depending on the type of disease and specific contexts. The findings align with that of a study conducted by Bener and Mohammad, which reported significantly increased risks for diabetes (OR = 2.88, 95% CI: 1.73–4.79, *P* < 0.001), heart diseases (OR = 2.89, 95% CI: 1.73–4.79, *P* < 0.001), cancer (OR = 5.18, 95% CI: 2.62–10.25, *P* < 0.001), and asthma (OR = 4.54, 95% CI: 2.45–8.41, *P* < 0.001).^19^ Similarly, in Saudi Arabia, several studies highlighted an increased risk of type 2 diabetes (T2D) in individuals from consanguineous unions compared to those from non-consanguineous unions. Alzahrani et al reported ORs of 1.151 and 1.476 for marriages between paternal and maternal first cousins, respectively.^20^ Additionally, Gosadi et al found an earlier onset of T2D and higher fasting blood glucose levels among CM,^21^ while Alsuhaimi and Albalawi confirmed that consanguinity is a significant risk factor for T2D, particularly in Arab countries.^22^

 Moreover, elevated frequencies of various multifactorial illnesses were noted within the consanguineous cohort in Bangladesh, encompassing bronchial asthma and renal disorders. Out of 84 children, 57 belonged to the consanguineous group exhibiting comorbidities.^23^ Longitudinal investigations performed in the Adriatic islands of Croatia highlighted a favorable correlation between consanguinity and a diverse spectrum of common disorders, particularly coronary diseases, high blood pressure, strokes, cancer, and asthma.^24^ In India, Bhasin and Kapoor demonstrated that the descendants of consanguineous couples have a nearly threefold higher risk of cardio-metabolic diseases. Their findings indicated a significant rise in the prevalence of NCDs, especially heart diseases (OR = 2.65, 95% CI: 1.02–6.85, *P*= 0.044), diabetes (OR = 2.44, 95% CI: 1.26–4.76, *P*= 0.009), and high blood pressure (OR = 2.62, 95% CI: 1.39–4.94, *P*= 0.003)^25^. These findings are in line with those of a study conducted in two Moroccan communities, which revealed significantly increased risks for CVD (OR = 2.411, 95% CI: 1.392–4.177, *P* = 0.002), diabetes (OR = 1.954, 95% CI: 1.131–3.375, *P*= 0.016), and cancer (OR = 2.102, 95% CI: 1.084–4.077, *P* = 0.026) among consanguineous individuals.^26^

 While these findings corroborate the results of several international studies. Some studies reported contrasting observations, particularly concerning certain types of cancer. Studies performed within the Arab community in Israel reported no association between consanguinity and the prevalence of NCDs, such as diabetes, myocardial infarction, or bronchial asthma^7^. This conclusion is supported by other studies that found no significant link between consanguinity and multifactorial disorders.^4^ Bener et al revealed variations based on cancer type. Consanguineous individuals showed an increased risk of leukemia, lymphoma, colorectal cancer, and prostate cancer but a reduced risk of other cancers, such as breast, skin, thyroid, and female genital cancer.^27^ The results of North African populations present a nuanced picture regarding the relationship between consanguinity and breast cancer (BC) risk. Two Tunisian studies suggested that consanguinity might have a protective effect against BC, particularly among women over 50 years old.^28,29^ This protective effect could be attributed to the absence of major risk factors in these populations or the potential role of recessive alleles in modifier genes.

 In contrast, the findings of a Moroccan study demonstrated no significant association between consanguinity and BC risk, even in families with a high predisposition to the disease.^30^ These findings conform to a broader conclusion drawn from a population-based case-control study, indicating that parental consanguinity does not appear to increase the risk of BC among Arab women. This suggests that other factors, rather than consanguinity, may play a more prominent role in determining BC risk in these populations.^31^ These discrepancies could be attributed to several factors, including differences in methodologies, the genetic diversity of studied populations, lifestyle influences, and complex interactions between genetic and environmental factors.

 Thus, the influence of consanguinity on multifactorial disorders in humans, especially in common diseases such as CVDs, cancer, diabetes, and asthma, remains unclear and subject to debate. These disorders typically involve multiple factors, with a complex etiology and suspected multifactorial transmission within numerous families. Certain highly susceptible genes might play a pivotal role in these diseases, and if these rare genes are transmitted recessively, consanguinity could be a determining factor.^4,18^ Some researchers suggest that consanguinity might impact various complex disorders in humans, particularly if the genetic component primarily involves numerous rare variants present in multiple genes, aligning with the hypothesis of common disease/rare variant.^18,24^ Given that most genetic variants associated with these diseases are partially recessive,^32^ consanguinity might increase the risk of disease by promoting homozygosity at multiple genetic loci, with subtle but adverse effects on homeostatic pathways.^24,33^

 These observations highlight the importance of conducting further research to better understand the effects of consanguinity on NCDs and the variations observed across different genetic and environmental contexts.

 This study has certain limitations. The cross-sectional design, while suitable for exploring associations and providing a foundation for future research, does not allow for establishing causality. Additionally, the reliance on self-reported data may introduce biases; however, efforts were made to mitigate this by cross-verifying information with medical records when available. Eventually, the lack of genetic analyses limits a deeper understanding of the biological mechanisms involved but highlights the need for future studies incorporating molecular approaches.

**Highlights**

- Non-communicable diseases had a higher rate in consanguineous marriages (CMs).
- Consanguineous participants had double the risk for cardiovascular diseases (CVDs).
- Participants from CM faced a fourfold higher risk of chronic kidney disease (CKD).
- Cancer risk was higher in CM, though not statistically significant.

## Conclusion

 Overall, more than a quarter of the population was consanguineous, and descendants of consanguineous parents exhibited higher rates of NCDs. These findings highlight that the increased incidence of these diseases is an additional disadvantage of CM, adding to the heightened risks of recessive genetic disorders. Acknowledging the high level of consanguinity and its adverse impacts on the health of future generations must be integrated into the country’s public health strategy. It is essential to implement appropriate genetic counseling, testing, and screening, along with an awareness program, particularly regarding genetic counseling. These measures aim to inform individuals about the drawbacks of consanguineous unions to reduce the prevalence of this practice and assist couples in making informed decisions in a society where traditions and family values are deeply ingrained.

## Acknowledgements

 We express our sincere appreciation to all individuals who took part in this study. Their valuable contributions greatly assisted in achieving our research objectives, even amid the challenges posed by the pandemic. We also express our appreciation to the healthcare professionals working in the primary health care services in the Settat province for their cooperation and support in ensuring access and data collection at health centers. This research owes its success to their significant contributions.

## Competing Interests

 The authors declare that they have no competing interests.

## Ethical Approval

 Researchers guarantee the confidentiality of the identity and data of participants. The information obtained will be exclusively used for research purposes. This study was conducted after obtaining approval from the Ethics Committee of the Moroccan Association for Research and Ethics (approval No. 3/REC/21). The research adhered to the ethical principles outlined in the 1964 Declaration of Helsinki and its subsequent amendments for all procedures involving human participants.

## Funding

 None.

## References

[1] Ministère de la Santé Publique (Maroc). Stratégie Nationale Multisectorielle de Prévention et de Contrôle des Maladies Non Transmissibles 2019-2029 [Internet]. 2019. Available from: https://www.sante.gov.ma/Documents/2019/02/Plan%20Strate%CC%81gique.pdf.

[2] Enquête Nationale sur les Facteurs de Risque Communs des Maladies Non Transmissibles 2017-2018: Rapport. Data for Health Initiative. Available from: https://data4healthlibrary.org/resources/enquete-nationale-sur-les-facteurs-de-risque-communs-des-maladies-non-transmissibles-2017. Accessed January 2, 2025.

[3] van Vliet J, Oates NA, Whitelaw E (2007). Epigenetic mechanisms in the context of complex diseases. Cell Mol Life Sci.

[4] Hamamy H, Antonarakis SE, Cavalli-Sforza LL, Temtamy S, Romeo G, Kate LP (2011). Consanguineous marriages, pearls and perils: Geneva international consanguinity workshop report. Genet Med.

[5] Jaber L, Halpern GJ, Shohat M (1998). The impact of consanguinity worldwide. Community Genet.

[6] Bener A, Alali KA (2006). Consanguineous marriage in a newly developed country: the Qatari population. J Biosoc Sci.

[7] Tadmouri GO, Nair P, Obeid T, Al Ali MT, Al Khaja N, Hamamy HA (2009). Consanguinity and reproductive health among Arabs. Reprod Health.

[8] Bittles AH, Hamamy HA. Endogamy and consanguineous marriage in Arab populations. In: Teebi AS, ed. Genetic Disorders Among Arab Populations. Berlin, Heidelberg: Springer; 2010. p. 85-108. 10.1007/978-3-642-05080-0_4.

[9] El-Mouzan MI, Al-Salloum AA, Al-Herbish AS, Qurachi MM, Al-Omar AA (2007). Regional variations in the prevalence of consanguinity in Saudi Arabia. Saudi Med J.

[10] Islam MM (2012). The practice of consanguineous marriage in Oman: prevalence, trends and determinants. J Biosoc Sci.

[11] Al-Kandari YY, Al-Kandari YY (2018). Consanguineous marriage and its relationship with sociocultural variables in urban and Bedouin geographical regions in Kuwait. Arab Humanit.

[12] El-Hazmi MA, Al-Swailem AR, Warsy AS, Al-Swailem AM, Sulaimani R, Al-Meshari AA (1995). Consanguinity among the Saudi Arabian population. J Med Genet.

[13] Hamamy H, Alwan S. The sociodemographic and economic correlates of consanguineous marriages in highly consanguineous populations. In: Kumar D, Chadwick R, eds. Genomics and Society. Oxford: Academic Press; 2016. p. 335-61. 10.1016/b978-0-12-420195-8.00018-5.

[14] Oniya O, Neves K, Ahmed B, Konje JC (2019). A review of the reproductive consequences of consanguinity. Eur J Obstet Gynecol Reprod Biol.

[15] El Goundali K, Chebabe M, Zahra Laamiri F, Hilali A (2022). The determinants of consanguineous marriages among the Arab population: a systematic review. Iran J Public Health.

[16] Ministère de la Santé, DPRF/DPE/SEIS. Enquête Nationale sur la Population et la Santé Familiale (ENPSF-2018) [Internet]. Available from: https://www.sante.gov.ma/Documents/2020/03/Rapport%20ENPSF%202018%202i%C3%A8me%20%C3%A9dition.pdf.

[17] Monographies de la Région. Site de la Direction Régionale de Casablanca-Settat [Internet]. Available from: https://www.hcp.ma/reg-casablanca/Monographies-de-la-Region_a2.html. Accessed January 2, 2025.

[18] Bittles AH, Black ML (2010). Evolution in health and medicine Sackler colloquium: consanguinity, human evolution, and complex diseases. Proc Natl Acad Sci U S A.

[19] Bener A, Mohammad RR (2017). Global distribution of consanguinity and their impact on complex diseases: genetic disorders from an endogamous population. Egypt J Med Hum Genet.

[20] Alzahrani SH, Alzahrani NM, Al Jabir FM, Alsharef MK, Zaheer S, Hussein SH (2021). Consanguinity and diabetes in Saudi population: a case-control study. Cureus.

[21] Gosadi IM, Goyder EC, Teare MD (2014). Investigating the potential effect of consanguinity on type 2 diabetes susceptibility in a Saudi population. Hum Hered.

[22] Alsuhaimi NA, Albalawi HI (2023). Consanguinity as a significant risk factor for diabetes mellitus: a systematic review. Int J Adv Res.

[23] Anwar S, Taslem Mourosi J, Arafat Y, Hosen MJ (2020). Genetic and reproductive consequences of consanguineous marriage in Bangladesh. PLoS One.

[24] Rudan I, Rudan D, Campbell H, Carothers A, Wright A, Smolej-Narancic N (2003). Inbreeding and risk of late onset complex disease. J Med Genet.

[25] Bhasin P, Kapoor S (2015). Impact of consanguinity on cardio-metabolic health and other diseases: findings from an Afro-Indian tribal community. J Community Genet.

[26] Goundali KE, Bouab C, Rifqi L, Chebabe M, Hilali A (2022). [Consanguineous marriages and their effects on non-communicable diseases in the Moroccan population: a cross-sectional study]. Pan Afr Med J.

[27] Bener A, El Ayoubi HR, Chouchane L, Ali AI, Al-Kubaisi A, Al-Sulaiti H (2009). Impact of consanguinity on cancer in a highly endogamous population. Asian Pac J Cancer Prev.

[28] Medimegh I, Troudi W, Omrane I, Ayari H, Uhrhummer N, Majoul H (2015). Consanguinity protecting effect against breast cancer among Tunisian women: analysis of BRCA1 haplotypes. Asian Pac J Cancer Prev.

[29] Troudi Cherif W, Uhrhummer N, Ben Ayed F, Bignon YJ, Sibille C, Benammar-Elgaaied A (2014). Does consanguinity protect against breast cancer in Tunisian population. Hereditary Genet.

[30] Elalaoui SC, Jaouad IC, Laarabi FZ, Elgueddari Bel K (2013). Low level of consanguinity in Moroccan families at high risk of breast cancer. Asian Pac J Cancer Prev.

[31] Denic S, Bener A, Sabri S, Khatib F, Milenkovic J (2005). Parental consanguinity and risk of breast cancer: a population-based case-control study. Med Sci Monit.

[32] Bittles AH, Neel JV (1994). The costs of human inbreeding and their implications for variations at the DNA level. Nat Genet.

[33] Ben Halim N, Ben Alaya Bouafif N, Romdhane L, Kefi Ben Atig R, Chouchane I, Bouyacoub Y (2013). Consanguinity, endogamy, and genetic disorders in Tunisia. J Community Genet.

